# Importance of lipid accumulation product index as a marker of CVD risk in PCOS women

**DOI:** 10.1186/s12944-015-0061-y

**Published:** 2015-06-24

**Authors:** Joelma Ximenes Prado Teixeira Nascimento, Maria Bethânia da Costa Chein, Rosângela Maria Lopes de Sousa, Alexsandro dos Santos Ferreira, Paula Andrea Navarro, Luciane Maria Oliveira Brito

**Affiliations:** Universidade Federal do Maranhão, São Luís, Maranhão Brazil; USP/Ribeirão Preto, São Paulo, São Paulo Brazil; Rua Boa Esperança, Cond. Bosque dos Pinheiros Qd. 03, C. 02 - Bairro: Turú, São Luís, Maranhão Brazil

**Keywords:** Polycystic ovary syndrome, Cardiovascular diseases, Lipid accumulation product

## Abstract

**Background:**

The polycystic ovary syndrome (PCOS) is considered the most common endocrine disease during the woman's reproductive life, with prevalence ranging from 5 to 10 % of women of reproductive age. There is a paucity of studies regarding the use of the lipid accumulation product (LAP) as a risk marker for the development of cardiovascular disease (CVD).

**Methods:**

A cross-sectional study was conducted on 78 women aged 18 to 42 years seen at University Hospital of Maranhão, with a diagnosis of polycystic ovary syndrome according to the Rotterdam criteria. The following variables of interest were recorded on a protocol form: sociodemographic and behavioral data, body mass index, waist circumference, fasting glucose, total cholesterol, triglycerides, low density lipoprotein cholesterol, high-density lipoprotein cholesterol, and systolic and diastolic blood pressure.

**Results:**

Logistic regression showed that, except for HDL, all cardiovascular risk markers presented a higher chance of being altered when the lipid accumulation product was above the cut off value of 37.9 cm.mmol/L.

**Conclusion:**

The lipid accumulation product seems to be sufficient to indicate a risk of cardiovascular diseases in women with polycystic ovary syndrome.

## Introduction

Polycystic ovary syndrome (PCOS) is an endocrine disorder which is characterized mainly by anovulation and hyperandrogenism. Clinical manifestations range from irregular menstrual cycles to the absence of menstruation associated with variable degrees of overweight and an increased risk of developing cardiovascular diseases, among others [1, 2]. PCOS is the most frequent gynecological endocrinopathy, affecting 5 to 10 % of premenopausal women [3]. In Brazil, Melo et al. [4] reported a prevalence of PCOS of 13.9 % in AGA women at birth and prevalence in SGA women at birth (30.2 %).

The criteria currently used for the diagnosis of PCOS are those established by the Rotterdam consensus [5], which defines the disease as the presence of two of the three following criteria: oligomenorrhea and/or anovulation, clinical and/or biochemical signs of hyperandrogenism, and polycystic ovaries on ultrasound (presence of 12 or more follicles measuring 2 to 9 mm in diameter or with a total volume > 10 cm^3^ in at least one ovary). The diagnosis is confirmed after the exclusion of other conditions that can cause chronic anovulation and androgen excess.

Cardiovascular disease (CVD) is one of the main public health problems, accounting for almost half of deaths in Europe and North America [6] and for 32 % of deaths in Brazil [7]. Obesity is a condition seen in 40 to 50 % of women with PCOS and the intensity of its symptoms is related to the degree of obesity [8]. Obesity alone contributes to the physiopathology of PCOS and is frequently associated with hyperinsulinemia [9], hypertriglyceridemia and reduced high-density lipoprotein cholesterol (HDL-c) [10], arterial hypertension, and type 2 diabetes mellitus [11]. These conditions are characterized by the redistribution of body fat mass, i.e., excess fat deposition in the abdominal region (androgenic obesity), which is associated with a higher CVD risk, particularly arterial hypertension and diabetes mellitus [12, 13].

The body mass index (BMI) is the most commonly used indicator to assess obesity. This indicator evaluates generalized obesity, but does not measure body fat distribution [12, 14]. In addition, BMI is a limited tool for the differentiation between body fat and lean mass and for the identification of their anatomical location or function of different fat deposits [15]. In this respect, a new index, called the lipid accumulation product (LAP), has been proposed.

Women with PCOS have been shown to be at an increased risk of developing CVD. This risk is even higher in the presence of obesity when the chances of an altered LAP are high [16]. Thus, the use of this index may render the evaluation of CVD risk more feasible, practical and less costly, especially at primary healthcare services in Brazil. However, few studies have investigated the application of this index to the population attending the Brazilian primary healthcare service the single health system (SUS). Other risk markers such as the Framingham Score [17] and the Reynold Risk Score [18] require a larger number of variables to assess cardiovascular risk, with a consequent increase in costs and the need for additional technologies.

The use of more objective diagnostic procedures at SUS is of particular importance when considering the increase in the incidence of CVD related morbidity and mortality observed during the nutrition transition, especially in women with PCOS. In view of the importance of the LAP index, the objective of the present study was to evaluate the association between this indicator and cardiovascular risk factors in women with PCOS, identify a cutoff point and compare it with changes in other cardiovascular markers. Our hypothesis is that LAP may be uncertain in this syndrome.

### Subjects and methods

An analytic cross-sectional study was conducted on 78 women aged 18 to 42 years with a diagnosis of PCOS according to the Rotterdam criteria [5]. The patients were seen at University Hospital (HU) of Maranhão (UFMA), between September 2010 and February 2012. Patients who do not contemplate such criteria were not sampled.

The non-probabilistic sampling was employed. All women were submitted to clinical examination, including the measurement of body weight, height, and WC. For WC, the lowest circumference between the last rib and iliac crest [19] was considered. The reference standards of the World Health Organization (1997) were used for the classification of nutritional status based on BMI: grade III thinness ≤ 16.0 kg/m^2^; grade II thinness: 16.0 – 16.9 kg/m^2^; grade I thinness: 17.0 – 18.4 kg/m^2^; eutrophy: 18.5 – 24.9 kg/m^2^; overweight: 25.0 – 29.9 kg/m^2^; grade I obesity: 30.0 – 34.5 kg/m^2^; grade II obesity: 35.0 – 39.9 kg/m^2^, and grade III obesity ≥ 40.0 kg/m^2^[19].

The LAP index was determined using the following equation: (WC [cm] - 58) x (TG [mmol/L]). The concentration of triglycerides in mmol/L used in the equation was obtained by multiplying their concentration in mg/dL by 0.0113 [15]. The following variables of interest were recorded on a protocol form: sociodemographic and behavioral data, BMI, WC, fasting glucose, total cholesterol (TC), triglycerides (TG), low density lipoprotein cholesterol (LDL-c), high-density lipoprotein cholesterol (HDL-c), and systolic (SBP) and diastolic blood pressure (DBP).

The more rigorous guidelines of the International Diabetes Federation (IDF) were used to establish cut off values of CVD risk, which is defined by the presence of three or more of the following variables: WC ≥ 80 cm considering ethnic background (since no specific data for South American women are available, cutoff values for European women were used); fasting glucose ≥ 5.6 mmol/L or a previous diagnosis of diabetes; TG ≥ 1.6 mmol/L or treatment of dyslipidemia; HDL-c < 1.2 mmol/L or treatment of dyslipidemia; SBP ≥ 130 mmHg or treatment, or DBP ≥ 85 mmHg. In addition, the Fourth Brazilian Guidelines for Dyslipidemias and Prevention of Atherosclerosis, which consider total cholesterol ≥ 5.1 mmol/L and LDL-c ≥ 4.1 mmol/L, were used [20, 21]. As for physical activity, inactivity was considered for those who did not perform any type of physical exercise on a regular basis for at least 3 times per week with at least 30 min. Smoking was confirmed when the habit was present - regardless of amount [21].

The patients were submitted to blood collection for biochemical determination of diagnosis of PCOS at the Centre for Clinical Research (CEPEC), University Hospital, UFMA, in the morning between 7 to 9 h, after fasting for 12 h, in the follicular phase (third the seventh day of the cycle) in those with regular menstrual cycles and any day in those without regularity.

For each patient, 20 ml was collected whole blood and stored under sterile vacuum tubes containing EDTA (for blood count) and serology (separating gel) without anticoagulant for biochemical and hormonal evaluation, using sterile equipment for such and disposable as the bio-security standards for biological material.

The processing of blood samples was begun at most 1 h after collection. The serum samples were stored in a freezer at −80 °C.

Fasting glucose and lipid profile were analyzed by enzymatic colorimetric method. LDL-c was calculated from the Friedewald formula: LDL-c = total cholesterol (HDL-c – TG / 5), as there was in the samples, we measured the upper TG 4.5 mmol/L. The biochemical measurements were performed with the Cobas 6000 equipment, Hitachi Hight-Technologies Corporation 24–14.Nish-shimbashi.1-chome.Minato-ku, Tokyo, Japan.

The Shapiro-Wilk test was used to determine whether the quantitative variables showed a normal distribution. The frequency distribution (absolute and relative) was performed for categorical variables. Quantitative CVD risk variables are expressed as the mean and standard deviation. A receiver operating characteristic (ROC) curve was used to identify the LAP index as a predictor of CVD risk as well as the cutoff point, estimating the highest sensitivity and specificity of this index. Logistic regression analysis was used to evaluate the association between cardiovascular risk markers (BMI, fasting glucose, total cholesterol, LDL-C, HDL-C, SBP, DBP, physical activity, and smoking habits) and the LAP index. The adjusted logistic regression was used to further control potential bias. It is noteworthy that there were no losses in the sample. The Stata® 12.0 program was used for statistical analysis and the Microsoft Office 2010® software for data tabulation. A level of significance of 5 % was adopted for all tests.

The study was approved by the Ethics Committee of HU-UFMA (Permit No. 349/11) and all participants signed a free informed consent form.

## Results

The sociodemographic and behavioral characteristics of the sample (n = 78). It is noteworthy that there was no loss in the sample (all participants contemplated the inclusion criteria). Noted a high frequency of age group 18–26 years (50.0 %), unmarried (65.4 %), unpaid (51.3 %), non-smokers (93.6 %) and physically active (79.5 %) (Table 1).

**Table 1:** Sociodemographic and behavioral characteristics of the sample

Variable	n (%)
Age (years)	
18 to 26	39 (50.0)
27 to 34	31 (39.7)
35 to 42	8 (10.3)
Marital status	
Single	51 (65.4)
Married	27 (34.6)
Occupation	
Paid	35 (48.7)
Unpaid	43 (51.3)
Smoking	
Yes	5 (6.4)
No	73 (93.6)
Physical inactivity^a^	
Yes	62 (79.5)
No	16 (20.5)

^a^As for physical activity, inactivity was considered for those who did not perform any type of physical exercise on a regular basis for at least 3 times per week with at least 30 min

**Fig. 1:**
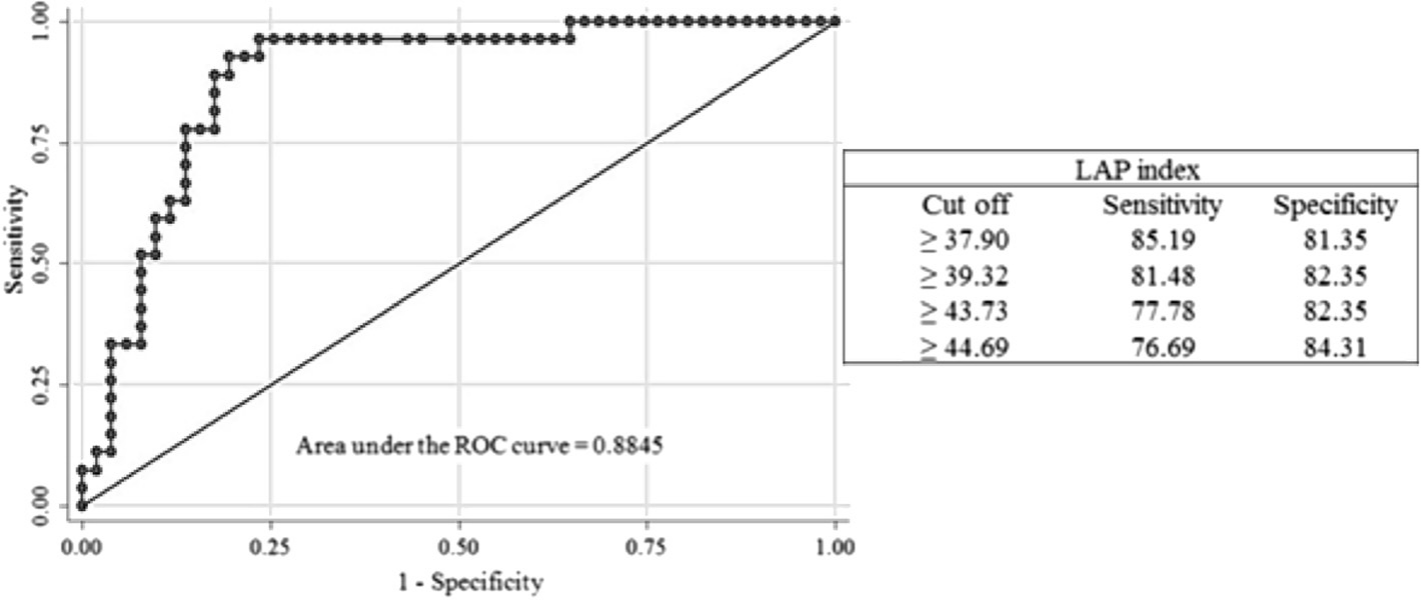
Distribution of lipid accumulation product (LAP index) values on the ROC curve of the sample. Lipid accumulation product

Considering the trade-off between specificity and sensitivity, the cut off point for the LAP index was ≥ 37.9 cm.mmol/L (sensitivity: 85.19 %, specificity: 81.35 %), corresponding to the value closer to 1.0 (best cutoff point), Fig. 1.

All cardiovascular risk parameters were associated with statistically the LAP index (*p* <0.05). Among women, 41 % had changes in LAP. Among obese 17.7 % had changes in the parameter, among those with high CC, 78.9 % among those with hyperglycemia, 83.3 %. Regarding women with lipid abnormalities, there was an association with changes in LAP index by more than 60 % in all analyzed fractions, Table 2.

**Table 2:** Cardiovascular risk factors associated with changes in LAP Index

Variable	LAP^a^		*p* – value*
	≥37.9 cm.mmol/L	<37.9 cm.mmol/L	
	(*n* = 32)	(*n* = 46)	
BMI^b^, n (%)			<0.001
Not obese	23 (85.2)	4 (14.8)	
Obese	9 (17.7)	42 (82.3)	
WC^c^, n (%)			<0.001
High	30 (78.9)	8 (21.1)	
Normal	2 (5.0)	38 (95.0)	
Fasting glucose, n (%)			0.028
High	5 (83.3)	1 (16.7)	
Normal	27 (37.5)	45 (62.5)	
Total cholesterol, n (%)			0.005
High	14 (66.7)	7 (33.3)	
Normal	18 (31.6)	39 (68.4)	
Triglycerides, n (%)			<0.001
High	16 (94.1	1 (5.9)	
Normal	16 (26.2)	45 (73.8)	
LDL-c^d^, n (%)			0.005
High	11 (73.3)	4 (26.7)	
Normal	21 (33.3)	42 (66.7)	
HDL-c^e^, n (%)			<0.001
Normal	20 (31.3)	44 (68.8)	
Low	12 (85.7)	2 (14.3)	
SBP^f^ (mmHg)			<0.001
Normotensive	6 (17.1)	29 (82.9)	
Not normotensive	26 (60.5)	17 (39.5)	
DBP^g^ (mmHg)			<0.001
Normotensive	7 (17.1)	34 (82.9)	
Not normotensive	25 (67.6)	12 (32.4)	

*Chi-square test; ^a^Lipid accumulation product (LAP index); ^b^Body mass index; ^c^waist circumference; ^d^low density lipoprotein; ^e^high density lipoprotein; ^f^systolic blood pressure; ^g^diastolic blood pressure

Among the normotensive, SBP and DBP as, 82.9 % was demonstrated-associated adjustments when the product of lipid accumulation, Table 2.

All women with an LAP index above the defined cut off point presented significantly more frequent altered mean values of the cardiovascular risk markers analyzed. The prevalence of women with an LAP index ≥ 37.9 cm.mmol/L was 41 % (Table 3).

**Table 3:** Distribution of cardiovascular risk markers according to cut off value of the lipid accumulation product (LAP index)

Variable	LAP^a^		*p*
	<37.9 cm.mmol/L	≥37.9 cm.mmol/L	
BMI^b^ (kg/m^2^)	23.1 ± 0.6	32.6 ± 0.9	<0.001
WC^c^ (cm)	74.6 ± 1.3	98.9 ± 1.7	<0.001
Fasting glucose (mmol/L)	4.7 ± 0.1	5.4 ± 0.3	0.006
Total cholesterol (mmol/L)	4.4 ± 0.1	5.2 ± 0.2	<0.001
Triglycerides (mmol/L)	0.9 ± 0.0	2.0 ± 0.1	<0.001
LDL-c^d^ (mmol/L)	2.6 ± 0.0	3.2 ± 0.1	<0.001
HDL-c^e^ (mmol/L)	1.3 ± 0.0	1.0 ± 0.0	<0.001
SBP^f^ (mmHg)	109.5 ± 1.7	122.8 ± 2.0	<0.001
DBP^g^ (mmHg)	70.0 ± 1.2	80.0 ± 1.4	<0.001
Total, n (%)	46 (59)	32 (41)	

Values are the mean ± standard deviation

^a^Lipid accumulation product (LAP index); ^b^body mass index; ^c^waist circumference; ^d^low density lipoprotein; ^e^high density lipoprotein; ^f^systolic blood pressure; ^g^diastolic blood pressure

The adjusted logistic regression was used to further control pontenciais bias. The test showed that, except for HDL-c, all cardiovascular risk markers presented a higher chance of being altered when the LAP was above the cutoff value of 37.9 cm.mmol/L. The other variables significantly increased (*p* <0.05) the LAP at least 8.4 times (CI > 1.0), except for HDL-c that was associated with a reduction in LAP (IC: 0.008 to 0.56), according to Table 4.

**Table 4:** Association between biases concerning cardiovascular system risk indicators and biases in the lipid accumulation product (LAP index) through adjusted logistic regression from the female population with polycystic ovary syndrome

Altered Variables	LAP^1^			
	Odds	Confidence Interval	Z	*P* value
BMI^2^ ≥ 30 kg / m^2^	32.6	6.4 – 163.8	4.23	<0.0001
HDL-c^3^ 1.2 mmol/L	0.06	0.008 – 0.56	−2.50	0.012
DBP^4^ ≥ 80 mmHg	8.4	1.86 – 37.9	2.77	0.006

^1^Lipid accumulation product (LAP index); ^2^body mass index; ^3^low density lipoprotein; ^4^diastolic blood pressure.

Were not included in the logistic regression model the variables waist circumference and triglycerides by these directly influence the value of the LAP.

The variables: BMI, fasting glucose, total cholesterol, LDL-C, HDL-C, SBP, DBP, physical activity and smoking habits were analyzed both in logistic regression models unadjusted, as in adjusted.

Before of adjust, only BMI and HDL-C showed statistically significant association with the LAP index, after adjustment, BMI and DBP were associated with a higher risk of changes in LAP and HDL-C to a reduction of changes in marker.

## Discussion

Cardiovascular risk parameters, were associated in women with abnormal LAP index. By comparison, women with such showed parameter higher measurements of BMI, CC, fasting glucose, total coleterol, triglycerides, LDL-c, SBP and DBP, as well as lower HDL-c.

Obesity marked by BMI and increased of DBP were associated with an increased risk of changes in LAP (above 8 times). The increase in HDL-c was associated with prevention for such change.

The aim of this study was to evaluate the association between LAP index and cardiovascular risk factors in women with PCOS. The results support the idea that women with PCOS who have the LAP index above the cut off suggested in this work showed the largest changes in mean CVD risk markers analyzed.

Even with a small sample in a cross-sectional study, considered a limitation in our study, the statistical analysis allowed away the null hypothesis. Still, some of the highlights in the study are the making of laboratory tests and the use of both anthropometric and blood pressure direct measurement which shall not be obtained through self-reference.

The adoption of low cost risk determining factors and also with fast responses are of great help towards treatment, tracking and early intervention which explains the reason why such marker contributes to the early diagnosis of the main cause for women’s mortality in the world.

In this study, women with PCOS, concentrated in the age group between 18 and 26 years, showing precociousness in the accumulation of risk factors for CVD. Revealing that this age group, an appropriate time for intervention in the development of CVD. Physical inactivity is an important cardiovascular risk factor [22, 23]. The highest percentage of physical inactivity in this study may favour the development of CVD especially when associated with smoking [21]. However, this habit often in the sample was low compared to the study of Junior Gil et al. [24].

The average age in our study was 26.3 years and the BMI was 27.01 kg/m^2^ with LAP index cutoff rate at ≥ 39.32 cm.mmol/L (81 % sensitivity and 82 % specificity) on CVD risk with a prevalence of 38.5 %; yet Wiltgen et al. [25] who evaluated LAP index in women with PCOS with an age average of 20.6 years and BMI average of 29.5 kg/m2 have come to a cut off rate of 37.87 cm.mmol/L (84 % sensitivity and 79 % specificity) thus, higher rates than ours. That may be explained due to the fact that a control case was carried out where the whole PCOS group had hyperandrogenism with high levels of testosterone. Nonetheless, it is controversial that hyperandrogenism by itself be considered a higher risk factor of CVD.

The combination of anovulation and hyperandrogenism translates PCOS in its classical form which shows the adverse metabolic phenotype of the syndrome. Such phenotype includes visceral obesity and resistance to insulin as well as a manifold of other traditional cardiovascular risk factors especially inflammation, glucose and dyslipidemia metabolism disorders [26].

The resulting risk increase of CVD may affect both lean women and obese ones. The subjacent mechanisms to the increase in cardiovascular risk within the PCOS context may include not only metabolic alterations, but also hormonal factors, hyperandrogenism in particular. Nevertheless, the consequences in terms of cardiovascular morbidity remain questionable due to the drawbacks in carrying out long term prospective studies aiming at identifying potential factors from those clinical results [26].

In this line of avaliation, 167 PCOS patients compared to 102 controls were evaluated and such study aimed at identifying coronary instability and diabetes mellitus type 2 through the frequency of CD4 (+) CD28 (null) lymphocytes that express the level of involvement by these morbidities. It has been concluded that there is a positive association between the frequency of CD4 (+) CD28 (null) lymphocytes in PCOS patients when they are compared to the control [27] showing that PCOS is a risk factor in the development of CVD.

For example it has been a study conducted with 139 women with PCOS aiming at evaluating both clinical and endocrine pictures as well as cardiovascular disease risk among the different PCOS phenotypes evaluated BMI values, series levels of follicle-stimulating hormones (FSH), luteinizing hormone (LH), progesterone, estradiol, testosterone, dehydroepiandrosteronesulphate (S-DHEA), fasting glucose, low density lipoprotein (LDL-C), total cholesterol, high density lipoprotein (HDL-C), C-reative protein, insulin, sensitivity to insulin and thickness of the carotid inner layer has found that the hyperandrogenic phenotype has lower risk of cardiovascular disease in comparison to other phenotypes [28].

Taking into account that the risk of cardiometabolic disorders may be partly determined by the employed definition of PCOS, clinical and epidemiological studies support the need to identify women with PCOS to determine the risk of cardiometabolic diseases and thus prevent and/or treat its serious consequences [29].

Women with PCOS associated with the presence of obesity, smoking habits, dyslipidemia, high blood pressure, intolerance to glucose and subclinical vascular disease are at risk whereas those with metabolic syndrome and/or diabetes mellitus type 2 pose high risk of CVD. It is therefore recommended that those women manage their lifestyles in order to seek the primary prevention of CVD [30].

Following the above findings with different design, prospective study, conducted at the Endocrinology, Diabetes and Metabolic Diseases Clinic at the University Center of Sarajevo, 50 patients diagnosed with PCOS in accordance with the Rotterdam criteria – ESHRE [5] were divided into two groups according to their BMI and had the following parameters evaluated: anthropometric indices (WC, height and weight), BMI, TG, resistance to insulin and the LAP index. Results showed that women with PCOS and BMI ≤ 24.9 kg/m^2^ were significantly different from those with BMI > 25 kg/m^2^ in body weight, WC and TG. Besides the LAP index having been a marker to differentiate women who were resistant to insulin from those who were not with a cut off value of 17.91 cm.mmol/L, the women group with BMI > 25 kg/m^2^ showed higher values of the LAP index. We may conclude that the LAP index is a marker to differentiate resistance to insulin in women with PCOS [31].

It was observed that prevalence of obesity and resistance to insulin in women with PCOS is significantly higher when compared to the population in general making the LAP index a new, cheap and accessible predictor of the metabolic syndrome both to the general population and women with PCOS [31].

In relation the area found under curve ROC (0.8845) shows that the LAP index presents satisfactory performance in those circumstances as its area above 0.70 translates a test with adequate diagnosis [32].

When screening patients under risk to develop CVD it is important that diagnostic tests present optimum sensitivity to detect disease or alterations in apparently healthy people (asymptomatic patients) [33]. Different studies indicated that the LAP index may be used as a screening marker when identifying patients who are likely to be characterized with the atherogenic metabolic triad: fasting hyperinsulinemia, hyperapolipo protein B and high proportion of small LDL-c particles [15, 16, 25, 34]. Notwithstanding, the LAP index discriminatory reach to identify patients with cardiometabolic risk profile may be compared to those of IDF and National Cholesterol Education Program – Adult Treatment Panel III (NCEP-ATP III) [20, 35] as it shows sensitivity, specificity [25] and low cost to identify people under higher cardiovascular risk.

Indeed, it is important to determine such index to evaluate women with PCOS once BMI alone is not capable of marking the fat deposition characteristic in the body [15]. It is doubtless that women with PCOS show high prevalence of central obesity [36, 37]. Studies demonstrate that 50 to 60 % of those patients present abdominal obesity regardless of their BMI [36–39].

Clinical practice and literature data have shown a high prevalence of cardiovascular risk indicators in women with PCOS [36, 37]. Since obesity is an independent risk factor of CVD [39], in our study the prevalence of obesity was 34.6 % with PCOS and its control is fundamental to improve clinical and biochemical alterations [13, 38]. Another variable used was WC, which is an indicator of abdominal adiposity and is widely used as a predictor of cardiovascular risk [39]. The frequency of increased WC was 41 %, a finding indicating its association with this syndrome [37].

Another risk factor for CVD already established is HDL-c levels below the recommended limit, irrespective of LDL-c values [10]. The percentage of women with reduced HDL-c observed in the present study was higher than that reported by Apridonidze et al. [40] who detected low HDL-c levels in 49 % of women with PCOS. A recent study including 406 patients with PCOS and 342 control women aged between 17 and 40 within a certain population in Southeast Asia between 2006 and 2011, a prevalence of 52.96 % of PCOS patients with dyslipidemia was found, which was around twice as much as in the controls, 28.95 %. The most common dyslipidemia components in PCOS patients were lowered levels of HDL-c (41.13 %) and an increase in TG (24.14 %). The frequency of metabolic syndrome in PCOS patients was 25.62 % which is more than five times higher when compared to the controls. The two main risk factors were the increase in waist circumference and low levels of HDL-c [41].

Obese women with PCOS present high SBP and DBP, suggesting that arterial hypertension is a late sequel of the stimulatory effects of hyperinsulinemia on the sympathetic nervous system and vascular smooth muscle [42]. In our study, the frequency of women with altered SBP and DBP was 14.1 %. In a case–control results with 60 women aged between 18 and 45 where 42 women with PCOS and 18 controls, observed no differences between the metabolic parameters and resistance to insulin in both groups. Comparing those parameters (metabolic and resistance to insulin) with 24-h blood pressure monitoring in the ambulatory (MAPA) it was shown that the only correlating parameter was the BMI, regardless of PCOS diagnosis. Such study observed that PCOS women did not show higher levels of blood pressure, glycemia, HDL-c, TG, resistance to insulin and BMI in comparison to the control group. However, women presented correlation among PCOS, blood pressure and BMI, suggesting that obesity is a main factor involved in blood pressure changes in those women [43].

Therefore, the use of anthropometric indices for the diagnostic evaluation of central obesity seems to be more adequate in this subgroup of women [13]. Using the cut off identified here, the LAP index may be a more accessible and more accurate tool to identify cardiovascular risk in women with PCOS and can be adopted and included in clinical practice by SUS on a regular basis since it is easy to be obtained and represents lower screening costs concerning those women under metabolic risk.
